# Substance Use and Suicide Attempts in Adolescents: A Comparative Analysis of Clinical and Psychosocial Risk Factors

**DOI:** 10.3390/children13020186

**Published:** 2026-01-29

**Authors:** Mustafa Tolga Tunagur, Elif Merve Kurt Tunagur

**Affiliations:** 1 Department of Child and Adolescent Psychiatry, Faculty of Medicine, Sakarya University, Serdivan 54290, Türkiye; 2 Department of Psychiatry, Faculty of Medicine, Çanakkale Onsekiz Mart University, Çanakkale 17020, Türkiye

**Keywords:** suicide, adolescent, substance-related disorders, risk factors, cannabinoids

## Abstract

**Highlights:**

**What are the main findings?**

- Female sex and high-frequency polysubstance use, particularly involving cannabinoids and stimulants, strongly differentiate adolescents with suicide attempts from those without.
- Addiction severity and functional impairment, rather than craving intensity alone, are closely associated with suicide attempt history.

**What are the implications of the main findings?**

- Synthetic cannabinoid and stimulant use should be systematically screened in suicide risk assessments among substance-using adolescents.
- Prevention and treatment strategies should be trauma-informed, gender-responsive, and focused on the psychosocial consequences of severe substance use.

**Abstract:**

**Background/Objectives**: This study aimed to compare clinical and psychosocial characteristics of substance-using adolescents in Türkiye with and without a history of suicide attempts to identify distinguishing risk factors. **Methods**: A retrospective analysis was conducted using medical records from 140 adolescents (aged 13–18) treated at a specialized Child and Adolescent Substance Use Center in Türkiye between March 2023 and February 2025. Sociodemographic, clinical, and substance use data were collected. Group comparisons were performed using chi-square and *t*-tests. Logistic regression was used to identify predictors of suicide attempt history. **Results**: Among the sample, 52 adolescents (37.1%) had a history of suicide attempts. Compared to their counterparts, the substance-using adolescents were more likely to be female (73.1%) and have histories of psychiatric hospitalization, institutional care, criminal behavior, and polysubstance use (*p* < 0.05). High-frequency use (≥3 days/week) of methamphetamine, stimulants, cannabinoids, and alcohol was significantly more common in this group (all *p* < 0.01). The Addiction Profile Index–Adolescent form (API-A) scores indicated more severe addiction profiles. Using cross-validated LASSO and confirmatory logistic regression, female gender emerged as the only robust independent predictor of suicide attempt history (OR = 6.84). **Conclusions**: Adolescents with a history of suicide attempts exhibit more severe substance use, particularly involving cannabinoids, and greater psychosocial adversity. This distinct risk profile underscores the need for early, gender-sensitive, and multidimensional interventions.

## 1. Introduction

Adolescent substance use and suicidal behaviors represent significant public health challenges worldwide, substantially contributing to morbidity and mortality among youth. It is estimated that in 2021, more than 10 million adolescents worldwide were affected by substance use disorders (SUDs) [1]. Concurrently, suicide remains among the primary causes of adolescent death, profoundly impacting families and communities [2]. The frequent co-occurrence of these issues complicates treatment and highlights the need for comprehensive prevention strategies targeting both substance abuse and suicidal behaviors in adolescents [3,4].

Growing evidence consistently demonstrates a strong association between substance use and suicidal thoughts and behaviors among adolescents [5]. Epidemiological studies indicate that adolescents who use substances, such as alcohol, cannabis, stimulants, or opioids, are significantly more likely to experience suicidal ideation, plans, or attempts [4,5,6]. Indeed, substance use acts as an independent risk factor, and this relationship appears to be dose-dependent and multifaceted: heavy or polysubstance use carries especially high risk [7]. A recent study indicated that adolescents using five or more different substances had up to a 16-fold increase in the likelihood of suicidal behavior [8]. Substance misuse often co-occurs with underlying depression or other psychiatric disorders that independently elevate suicide risk, and substance use can further exacerbate these conditions [9]. Collectively, these findings underscore that adolescents who misuse substances represent a high-risk population for suicidality, requiring careful assessment and intervention in both community and clinical contexts [10].

Adolescent suicide risk arises from a combination of individual, familial, and contextual factors [11]. At the individual level, psychiatric disorders, particularly depression and anxiety, strongly predict suicidal behavior [12]. Previous suicide attempts are a critical indicator of future risk [13]. Personality traits such as impulsivity, aggression, and feelings of hopelessness further heighten vulnerability [2]. Additionally, early adverse experiences like childhood trauma, abuse, or neglect significantly increase both substance misuse and suicidal risk [13].

Family environments play a crucial role in adolescent suicidality. Dysfunctional family dynamics, such as parental conflict, family history of mental illness or substance abuse, and adverse socioeconomic conditions, are associated with increased suicide attempts among adolescents [2,13]. Beyond familial influences, broader contextual factors significantly influence suicide risk. Difficulties in academic settings, such as school failure or disciplinary issues, and problematic peer relationships, including bullying and social isolation, contribute significantly to adolescent suicidal behaviors. Moreover, experiences of bullying, cyberbullying, physical or sexual abuse, and legal troubles frequently precede suicidal attempts [2,14]. These stressors are especially common among substance-using adolescents who attempt suicide, suggesting that substance use and stressful environments jointly increase suicide risk [2,15].

Substance use, psychiatric symptoms, and psychosocial adversities do not act in isolation but interact in a dynamic and mutually reinforcing way [16]. Developmental psychopathology and stress–diathesis models suggest that emotional dysregulation, impulsivity, and adverse family and social environments together increase vulnerability to both substance misuse and suicidal behavior [17]. Empirical studies showing links between substance use severity, depression, trauma, and impaired family functioning support transactional models in which substance use both reflects and worsens underlying emotional and interpersonal difficulties [18,19]. These interconnected pathways underscore the need to examine clinical, psychosocial, and substance-related factors within an integrated framework rather than as separate risk domains [20,21].

Despite considerable research on adolescent substance use and suicidality, notable gaps remain, particularly regarding data from low- and middle-income countries (LMICs) [13]. Much existing knowledge is derived from high-income Western countries, limiting generalizability to other contexts. Recent systematic reviews highlight this disparity, noting that studies from LMICs, which account for a significant proportion of global adolescent suicides, remain relatively scarce [6]. Moreover, research often focuses predominantly on substances like alcohol and tobacco, neglecting others such as synthetic cannabinoids, stimulants, and opioids, which are increasingly prevalent among adolescents in diverse settings [22].

In Türkiye, a middle-income country with unique cultural and socioeconomic characteristics bridging Eastern and Western contexts, comprehensive research exploring adolescent substance use and suicidal behavior remains particularly limited. Preliminary reports indicate that intentional drug overdose is common among Turkish adolescents attempting suicide [23,24]. Nevertheless, rigorous studies investigating associated risk factors and substance use patterns are scarce. This shortage of locally documented evidence hampers the development of culturally appropriate prevention and intervention strategies tailored to youth in Türkiye and similar contexts [23,25,26].

While prior research establishes the broad relationship between adolescent substance use and increased suicidality, a detailed exploration of distinguishing factors remains insufficient. Identifying risk profiles unique to suicidal adolescents is essential for improving assessment and targeting interventions effectively. Addressing these gaps, the current study aimed to investigate differences between substance-using adolescents with and without a history of suicide attempts. This study aimed to investigate differences in sociodemographic features, clinical profiles, and patterns of substance use between the two groups at a treatment center specializing in adolescent substance use in Türkiye. We hypothesize that adolescents with a history of suicide attempts will demonstrate significantly higher levels of psychiatric comorbidity, familial dysfunction, and frequency and severity of substance use compared to adolescents without a history of suicide attempts.

## 2. Materials & Methods

### 2.1. Sample

This study was conducted at a Child and Adolescent Substance Use Treatment Center (ÇEMATEM) in Türkiye. The medical records of adolescents who presented to the ÇEMATEM outpatient clinic between March 2023 and February 2025 were retrospectively reviewed. Sociodemographic characteristics, clinical data, substance use profiles, and suicide-related information of the adolescents were systematically collected. Information regarding the history of suicide attempts was obtained through clinical interviews with both adolescents and their parents. This information was then corroborated by reviewing digital medical records and previous hospital documents, thus enabling cross-validation of the data based on their self-reports. As this was a retrospective chart review, ethical approval was obtained prior to data access and analysis. The study was approved by the University’s Health Sciences Scientific Research Ethics Committee on 20 September 2025 (Approval No: E.513106-458). No patient records were reviewed before this approval was granted, in accordance with the Declaration of Helsinki.

Inclusion criteria were: (i) age between 12 and 18 years, (ii) presentation to the ÇEMATEM outpatient clinic between March 2023 and February 2025, and (iii) availability of sufficient clinical and sociodemographic information in the medical records to assess substance use characteristics and history of suicide attempts.

Exclusion criteria were: (i) incomplete or missing key medical records, (ii) presence of an acute psychotic episode or severe cognitive impairment at the time of outpatient evaluation that precluded reliable clinical assessment, and (iii) severe or unstable medical conditions that could interfere with psychiatric evaluation.

### 2.2. ÇEMATEM Procedures

All cases at ÇEMATEM are evaluated through a standardized assessment protocol. Upon admission, each adolescent is first assessed by a counselor specialized in addiction. Sociodemographic information, personal and family psychiatric history, clinical status, history of suicide attempts, and substance use characteristics are recorded in a structured evaluation form. Individual and motivational interviews are conducted with the adolescent by the counselor. Subsequently, the adolescents complete standardized assessment tools related to substance use, such as the Addiction Profile Index—Adolescent Form and the Substance Craving Scale.

In addition, family interviews are conducted with the adolescent’s parents, and all interviews are documented using standardized forms. Once completed, these forms and assessment scales are reviewed by the center’s consulting child and adolescent psychiatrist. This child and adolescent psychiatrist implements semi-structured interview schedules and scales to assess psychiatric comorbidities. All follow-ups and motivational interviews are conducted by counselors under the supervision of the child and adolescent psychiatrist.

### 2.3. Measurements

The Addiction Profile Index—Adolescent Form (API-A): The Addiction Profile Index is a scale that evaluates the dimensions of addiction and measures the severity of addiction. The Addiction Profile Index adolescent form should be applied to the 15–18 age group known to use alcohol and substances. This form consists of 25 questions and 5 subscales: (1) substance use characteristics, (2) diagnosis, (3) impacts on life, (4) cravings, (5) motivation, in addition to a total score (severity of addiction). Additionally, there is both a self-report form and a practitioner form. The form can assess alcohol and non-alcohol substance use. In the original validation study, internal consistency coefficients (Cronbach’s α) ranged from 0.63 to 0.86 across subscales, with an overall α of 0.87, indicating good reliability [27]. In the present sample, the internal consistency of the total API-A score was also high (Cronbach’s α = 0.83), supporting the reliability of the measurements.

Substance Craving Scale: This scale is an adaptation of the Penn Alcohol Craving Scale for non-alcoholic substance abusers. This scale consists of five items assessing frequency, intensity, and intrusiveness of craving, perceived control over craving, and craving-related distress. Each item is scored between 0 and 6. The total score from the scale is between 0 and 30. For non-alcoholic substance abusers, the Cronbach’s alpha value of this scale was found to be 0.84 for the entire scale, and the item-total correlation values for each item ranged from 0.75 to 0.82. The scale has been adapted to Turkish [28].

Substance use frequency was assessed based on self-report and clinical records. Frequent use was operationalized as use on ≥3 days per week on average during the past year before the visit to the ÇEMATEM outpatient clinic. Consistent with previous studies, using three or more days a week was considered heavy use [29,30,31].

Background variables included age, sex, substance use-related hospitalization history, institutional care under state protection, history of running away from home, criminal history, and family history of substance use and criminality, all extracted from standardized clinical intake forms. Psychiatric hospitalization history referred exclusively to inpatient admissions for alcohol or substance use treatment; hospitalizations for other psychiatric conditions (e.g., mood or anxiety disorders) were not included.

### 2.4. Statistical Analysis

The skewness and kurtosis values and inspection of histograms recommended in the previous guideline were used to assess the normality of the distribution of continuous variables [32]. Descriptive statistics are presented as frequencies, percentages, means, and standard deviations. For normally distributed variables, group comparisons were conducted using Student’s *t*-test, and homogeneity of variances was evaluated with Levene’s test. Chi-square tests were used to analyze associations between categorical variables.

To identify robust predictors of suicide attempt history while minimizing overfitting and selection bias, least absolute shrinkage and selection operator (LASSO) regression was used for variable selection [33]. All candidate predictors (age, gender, institutional care under state protection, criminal history, alcohol use, stimulant use, and cannabinoid use) were entered simultaneously into a LASSO model with standardized predictors and an intercept. The regularization parameter λ was systematically varied from 0.01 to 2.00 in 0.01 increments and the optimal λ was selected using k-fold cross-validation. Variables with non-zero coefficients at the optimal λ were retained and subsequently entered into a conventional binary logistic regression model (enter method) to obtain odds ratios and 95% confidence intervals.

The number of days per week was used to express substance use frequency (days when substance use occurred at least once). Statistical significance was set at *p* < 0.05, and 95% confidence intervals are reported when appropriate. All analyses were conducted using IBM SPSS Statistics for Windows, Version 31.0 (Released 2026; IBM Corp., Armonk, NY, USA).

Post hoc power analyses were conducted using G*Power, Version 3.1.9.7 (Released 2020; Heinrich Heine University Düsseldorf, Düsseldorf, Germany) (logistic regression, two-tailed, α = 0.05). For the observed association between female sex and suicide attempt history (OR = 7.01; N = 140; π = 0.45), the achieved power was 0.99; for cannabinoid use (OR = 3.49; N = 140; π = 0.729), the achieved power was 0.89.

## 3. Results

Over a two-year period, a total of 143 adolescents presented to the ÇEMATEM. Three adolescents were excluded from the study: one due to acute psychosis and two due to incomplete clinical data. Of 140 adolescents, 55.0% (n = 77) were male and 45.0% (n = 63) were female. A history of suicide attempt was identified in 52 individuals. The most common method of suicide attempt was drug intake, reported in 25 cases (48.1%). This included both medications prescribed to the adolescents themselves and medications available in the household (e.g., analgesics, antibiotics, antidiabetics, and psychotropic agents belonging to family members), as documented in clinical interviews and medical records. The mean number of suicide attempts among those with a history was 1.8 ± 1.0 (range: 1–4).

Regarding the referral pathway to ÇEMATEM, 49 adolescents (35.0%) sought treatment voluntarily, 58 (41.4%) were referred due to family suspicion, 26 (18.6%) were brought in by state authorities, and 7 (5.0%) were admitted as part of a legal health precaution order.

In terms of educational status, 10 adolescents (7.1%) had completed primary school, 41 (29.3%) had graduated from middle school, and 89 (63.6%) were currently attending high school. Substance use within the school environment was reported in 60 cases (42.9%).

There was no significant difference in mean age between adolescents with and without a history of suicide attempt. However, the suicide attempt group included a significantly higher proportion of females compared to the non-attempt group (*p* < 0.001). Previous psychiatric hospitalization was significantly more common among those with a history of suicide attempt (*p* < 0.001). Similarly, running away from home (*p* = 0.005), history of institutional care under state protection (*p* = 0.002), personal criminal history (*p* = 0.020), and polysubstance use (*p* = 0.004) were all significantly more prevalent in the suicide attempt group. In contrast, family history of alcohol/substance use and criminal behavior did not significantly differ between the groups. A detailed comparison of sociodemographic and clinical variables is presented in Table 1.

Adolescents with a history of suicide attempt were significantly more likely to use methamphetamine (*p* = 0.009), stimulants (defined as methamphetamine and/or ecstasy; *p* = 0.007), cannabis (*p* = 0.017), any cannabinoid (including cannabis and synthetic cannabinoids; *p* = 0.001), and alcohol (*p* = 0.011) compared to those without such a history. Ecstasy (*p* = 0.059), synthetic cannabinoid (*p* = 0.091), pregabalin (*p* = 0.093), and inhalant use (*p* = 0.055) were more common in the suicide attempt group, though these differences did not reach statistical significance. Detailed substance use comparisons between the groups are presented in Table 2.

Adolescents with a history of suicide attempt reported significantly more frequent use (≥3 days per week) of methamphetamine, ecstasy, stimulants, cannabis, synthetic cannabinoids, cannabinoids (overall), and alcohol compared to those without such a history (all *p* ≤ 0.002). In particular, high-frequency methamphetamine use was observed in 75.0% of the suicide attempt group versus 26.5% of the non-attempt group (*p* < 0.001), and similar patterns were seen across other substances. Table 3 provides a detailed comparison of substance use frequency between groups.

While there were no significant group differences in age at first cigarette or substance use, craving intensity, or motivation levels, adolescents with a history of suicide attempt scored significantly higher on the API-A total score (*p* = 0.002), substance use characteristics (*p* = 0.001), clinical diagnosis severity (*p* = 0.008), and substance-related impact on life (*p* = 0.001). These findings suggest a more severe substance use profile among adolescents with a history of suicide attempt. Table 4 presents a detailed comparison of substance-related characteristics between the two groups.

Using cross-validated LASSO regression, sex was the only variable retained in the model at the optimal level of regularization. In contrast, the coefficients of institutional care, criminal history, alcohol use, stimulant use, and cannabinoid use were reduced to zero. These findings indicate that, among the candidate variables, sex represented the most stable and generalizable predictor of suicide attempt history.

In the subsequent confirmatory binary logistic regression model including the LASSO-selected predictor, female adolescents had significantly higher odds of reporting a lifetime suicide attempt compared with males (OR = 6.84, 95% CI = 3.17–14.75, *p* < 0.001; Table 5). The model explained 24.0% of the variance (Nagelkerke R^2^ = 0.240). Detailed results of the regression analysis are presented in Table 5.

## 4. Discussion

This study provides a comprehensive comparison of substance-using adolescents with and without a history of suicide attempts in a clinical setting in Türkiye. Consistent with prior research, our findings highlight that female gender, psychiatric hospitalization, institutional care, criminal history, and particularly polysubstance use are significantly more prevalent among adolescents who have attempted suicide. These results extend the evidence base by emphasizing these patterns within a middle-income country context, where such data have been scarce.

One of the key findings of this study was a significant difference in suicide attempts between female and male adolescents. Female adolescents were more common in the group that attempted suicide, while male adolescents were more common in the group that did not attempt suicide. This suggests that female adolescents with substance use problems may be more prone to suicide attempts, consistent with previous studies showing that female adolescents attempt suicide more frequently [34,35]. Our regression analysis further highlights the independent predictive value of female gender for suicide attempts, as emphasized in larger epidemiological studies [36]. This sex-specific vulnerability may be partly related to unmeasured interpersonal trauma, particularly sexual abuse, which is more prevalent among female adolescents and is strongly associated with both substance use and suicidal behavior [37]. In addition, family interaction patterns and emotional support, not captured by simple family history variables, may contribute to this increased risk and warrant further investigation in future prospective studies.

Polysubstance use emerged as a critical marker distinguishing adolescents with a history of suicide attempts. Substances such as methamphetamine, alcohol, cannabis, and notably synthetic cannabinoids and ecstasy were disproportionately used at high frequencies among this group, a trend consistently associated with increased impulsivity, mood instability, and suicidal behaviors [36,38,39]. Given the more severe psychiatric and behavioral effects reported with synthetic cannabinoids, the type of cannabinoid used may be particularly relevant in suicide risk assessment [40]. The elevated API-A scores further reflect greater substance use severity, adding weight to the evidence that more entrenched addiction profiles correlate with heightened suicide risk.

Furthermore, heavy methamphetamine use (≥3 days/week) was significantly more frequent among adolescents who attempted suicide. This finding highlights the strong association between stimulant exposure and suicidal behavior. In contrast, while craving scores did not differ significantly between groups, overall dependence severity (API-A total score) and its functional consequences, particularly the ‘Impact on Life’ domain, were significantly higher in the group that attempted suicide. This pattern suggests that suicidal behavior in youth is more closely linked to the psychosocial and functional burden of substance use than to craving or physiological dependence itself.

These results reinforce the established link between psychiatric comorbidities and suicide attempts among adolescents with substance use disorders [41,42]. The bidirectional interaction between emotional dysregulation, substance use as a maladaptive coping mechanism, and worsening psychiatric symptoms appears to amplify suicidal risk. Additionally, psychosocial risk factors—such as prior institutional care and legal system involvement—were significantly associated with suicide attempts, echoing findings that adverse early environments and systemic marginalization contribute to both substance use and suicidality [43,44]. Such vulnerabilities must be accounted for when designing targeted interventions.

Previous studies have shown that family dysfunction is a significant risk factor for suicidal behavior in adolescents [11]. Interestingly, no significant differences were found between adolescents with and without suicide attempts in terms of family-related factors, such as a family history of psychiatric disorders or substance use. Our results suggest that in this clinical sample of young people with substance use problems, personal factors may be more important than family history in distinguishing those who attempted suicide from those who did not. This may be because family adversities are so common in both groups that they reduce observable differences, or because information based on retrospective medical records may not fully reflect the severity of family problems.

Recent network-based and hierarchical models have also highlighted that the family environment functions primarily as a distal vulnerability factor. In contrast, proximal factors such as psychopathologies and addiction severity have shown stronger direct associations with suicide attempts, particularly in high-risk clinical samples [45]. Similarly, studies in clinical populations have reported that current psychopathology and functional impairment may outweigh family history in predicting suicidal behavior once a substance use disorder is established [46].

Nevertheless, the lack of group differences in family history does not mean that family processes are unimportant. Previous research has shown that protective family factors, such as emotional support, communication, and parental monitoring, can reduce suicide risk even in the presence of significant adversity [44,47]. Therefore, both vulnerability and protective family dynamics should be considered within a developmental framework.

Emerging literature further highlights the importance of integrated intervention models. Neurobiological research shows that early and sustained exposure to substances like synthetic cannabinoids can disrupt adolescent brain development, particularly in regions governing executive function and emotion regulation [43,48]. Simultaneously, family- and school-based interventions—particularly those emphasizing resilience, coping skills, and trauma-informed care—have demonstrated promising results in preventing both substance misuse and suicidal behaviors [36,49].

Despite its strengths, including a well-characterized clinical sample and standardized data collection, this study has limitations. The cross-sectional and retrospective nature of the data does not allow for the determination of the temporal or causal direction between substance use and suicide attempts. The relatively modest sample size (n = 140) may have limited statistical power and reduced the precision of effect size estimates. Additionally, the sensitivity of the estimated effect sizes may have decreased after accounting for multicollinearity and shared variance using LASSO-selected predictor logistic regression analysis. Moreover, the lack of a healthy control group restricts conclusions to substance-using adolescents and prevents comparisons with the general population. In addition, the odds ratio for sex was associated with a relatively wide confidence interval, reflecting limited precision of the estimate, likely due to the small number of males in the suicide attempt group. Another limitation is the lack of standardized measures of depression and anxiety. Although these symptoms are well-known predictors of adolescent suicidal behavior, they could not be systematically assessed due to the retrospective design and incomplete clinical records. As a result, the potential confounding effects of these symptoms have not been fully controlled, which may have led to an overestimation of substance-related associations. Nevertheless, the findings provide critical direction for targeted prevention and early intervention strategies in Türkiye and similar LMIC contexts.

## 5. Conclusions

Adolescents with a history of suicide attempts exhibit more severe substance use profiles, marked by high-frequency, polysubstance consumption—particularly involving synthetic cannabinoids and stimulants. However, female gender emerged as the only stable and strongest independent predictor of suicide attempt history. These findings highlight the importance of gender-sensitive and trauma-focused prevention and treatment strategies in adolescents with substance use disorders. Future longitudinal research should focus on the psychological and motivational pathways linking substance use to suicidal behavior, such as self-medication of depressive and trauma-related symptoms versus sensation-seeking and impulsivity-driven use. Clarifying these motivational profiles may help identify distinct risk mechanisms and inform more personalized, developmentally sensitive prevention and intervention strategies. In parallel, implementation studies evaluating culturally adapted, community-based intervention strategies are urgently needed to reduce suicide risk among vulnerable adolescents.

## Figures and Tables

**Table 1:** Comparison of sociodemographic and clinical characteristics between adolescents with and without a history of suicide attempt.

Variables	Suicide Attempt Group (n = 52)	Non-Suicide Attempt Group (n = 88)	Statistics	*p*
	Mean (SD)	Mean (SD)	*t*	
Age, year	16.2 (1.5)	16.1 (1.3)	0.499	0.618
	**n (%)**	**n (%)**	**χ^2^**	
Gender			26.350	<0.001
Male adolescent	14 (26.9)	63 (71.6)		
Female adolescent	38 (73.1)	25 (28.4)		
Psychiatric Hospitalizations ^a^	31 (59.6)	25 (28.4)	13.263	<0.001
Running away from home	39 (75.0)	45 (51.1)	7.756	0.005
Institutional care under state protection	22 (42.3)	16 (18.2)	9.620	0.002
Criminal history	33 (63.5)	38 (43.2)	5.378	0.020
Family history of alcohol/substance use	32 (61.5)	47 (53.4)	0.879	0.349
Family history of criminal history	29 (55.8)	42 (47.7)	0.846	0.358
Polysubstance use	43 (82.7)	52 (59.1)	8.347	0.004

SD: Standard deviation. ^a^ History of inpatient treatment for alcohol and/or substance use disorders.

**Table 2:** Comparison of substance use characteristics between adolescents with and without a history of suicide attempt.

Substance Use	Suicide Attempt Group (n = 52)	Non-Suicide Attempt Group (n = 88)	*χ* ^2^	*p*
	n (%)	n (%)		
Methamphetamine use	32 (61.5)	34 (38.6)	6.880	0.009
Ecstasy use	28 (53.8)	33 (37.5)	3.552	0.059
Stimulant use (any)	38 (73.1)	44 (50.0)	7.173	0.007
Cannabis use	42 (80.8)	54 (61.4)	5.711	0.017
Synthetic cannabinoid use	37 (71.2)	50 (56.8)	2.855	0.091
Any cannabinoid use	46 (88.5)	56 (63.6)	10.186	0.001
Alcohol use	48 (92.3)	66 (75.0)	6.475	0.011
Pregabalin use	20 (38.5)	22 (25.0)	2.821	0.093
Inhalant use	25 (48.1)	28 (31.8)	3.673	0.055

**Table 3:** Comparison of substance use frequency between adolescents with and without a history of suicide attempt.

Substance Use Frequency	Suicide Attempt Group	Non-Suicide Attempt Group	*χ* ^2^	*p*
	n (%)	n (%)		
Methamphetamine use (n = 66)			15.529	<0.001
<3 days/week	8 (25.0)	25 (73.5)		
≥3 days/week	24 (75.0)	9 (26.5)		
Ecstasy use (n = 61)			10.913	<0.001
<3 days/week	15 (53.6)	30 (90.9)		
≥3 days/week	13 (46.4)	3 (9.1)		
Any stimulant use (n = 82)			12.451	<0.001
<3 days/week	18 (47.4)	37 (84.1)		
≥3 days/week	20 (52.6)	7 (15.9)		
Cannabis use (n = 96)			10.736	0.001
<3 days/week	22 (52.4)	45 (83.3)		
≥3 days/week	20 (47.6)	9 (16.7)		
Synthetic cannabinoid use (n = 87)			13.839	<0.001
<3 days/week	11 (29.7)	35 (70.0)		
≥3 days/week	26 (70.3)	15 (30.0)		
Any cannabinoid use (n = 102)			9.593	0.002
<3 days/week	13 (28.3)	33 (58.9)		
≥3 days/week	33 (71.7)	23 (41.1)		
Alcohol use (n = 114)			19.989	<0.001
<3 days/week	18 (37.5)	52 (78.8)		
≥3 days/week	30 (62.5)	14 (21.2)		

**Table 4:** Comparison of substance profiles between adolescents with and without a history of suicide attempt.

Variables	Suicide Attempt Group (n = 52)	Non-Suicide Attempt Group (n = 88)	*t* ^a^	*p*
	Mean (SD)	Mean (SD)		
Age at first cigarette use	12.0 (1.9)	11.8 (2.2)	0.677	0.500
Age at first substance use	14.1 (1.5)	14.0 (1.6)	0.216	0.830
Substance craving scale	15.5 (9.0)	13.7 (8.4)	0.820	0.415
API-A total score	13.0 (4.1)	10.0 (4.3)	3.272	0.002
Substance use characteristics	3.4 (1.4)	2.4 (1.4)	3.398	0.001
Diagnosis	13.7 (6.4)	10.3 (6.0)	2.733	0.008
Impact on life	16.5 (6.7)	11.4 (6.9)	3.374	0.001
Intensity of craving	2.7 (1.4)	2.2 (1.9)	1.337	0.185
Motivation level	3.1 (1.2)	2.9 (1.5)	0.795	0.429

API-A: Addiction Profile Index—Adolescent Form. SD: Standard deviation. ^a^ Homogeneity of variances was assessed using Levene’s test. All Levene’s F values were >0.05 and degrees of freedom were 138.

**Table 5:** LASSO-selected predictor and confirmatory logistic regression analysis predicting suicide attempt history among adolescents.

Variables ^a^				95% CI		
	β	Wald	OR	Lower	Upper	*p*
Gender	1.923	24.067	6.840	3.173	14.746	**<0.001**
Constant ^b^	0.543	7.669	1.721	-	-	**0.006**

β: Regression coefficient; CI: Confidence interval; OR: Odds ratio. Reference point: Non-suicide attempt group. Reference category for gender is male. ^a^ Predictors were selected using least absolute shrinkage and selection operator (LASSO) regression with cross-validation and standardized predictors. Variables with non-zero coefficients at the optimal penalty parameter (λ) were retained and entered into the confirmatory binary logistic regression model (enter method). ^b^ Adjusted *R*^2^ = 0.240.

## Data Availability

The data supporting the findings of this study are available upon request from the corresponding author. The data are not publicly available due to privacy or ethical restrictions.
